# Ocular Distribution of the Renin-Angiotensin-Aldosterone System in the Context of the SARS-CoV-2 Pandemic

**DOI:** 10.1155/2022/9970922

**Published:** 2022-02-03

**Authors:** Ali Abid, Muhammad Azaan Khan, Brendon Lee, Andrew White, Nicole Carnt, Sana Arshad, Chameen Samarawickrama

**Affiliations:** ^1^University of New South Wales, Australia; ^2^University of Sydney, Australia

## Abstract

The COVID-19 pandemic has resulted in an unprecedented impact on global health, economy, and way of life. SARS-CoV-2, the virus responsible for the disease, utilizes the ACE2 receptor found on host cells to mediate entry, replication, and infection. Numerous studies have elucidated the presence of many components of the renin-angiotensin-aldosterone system (RAAS) in the eye, including the ACE2 receptor. Considering this, and the anatomical vulnerability that the exposed ocular surface offers with its interconnectedness to the respiratory system, there is a theoretical risk of pathogen entry from the ocular route as well as the development of COVID-19-associated eye disease. Despite this, the actual epidemiological data demonstrates low ocular symptoms, possibly due to differing ACE2 receptor expression across age, ethnicity, and sex coupled with the protective properties of tears. We summarize the current literature on ocular RAAS with specific focus on the ACE2 receptor and its interplay with the SARS-CoV-2 virus.

## 1. Methods

**Figure 1:**
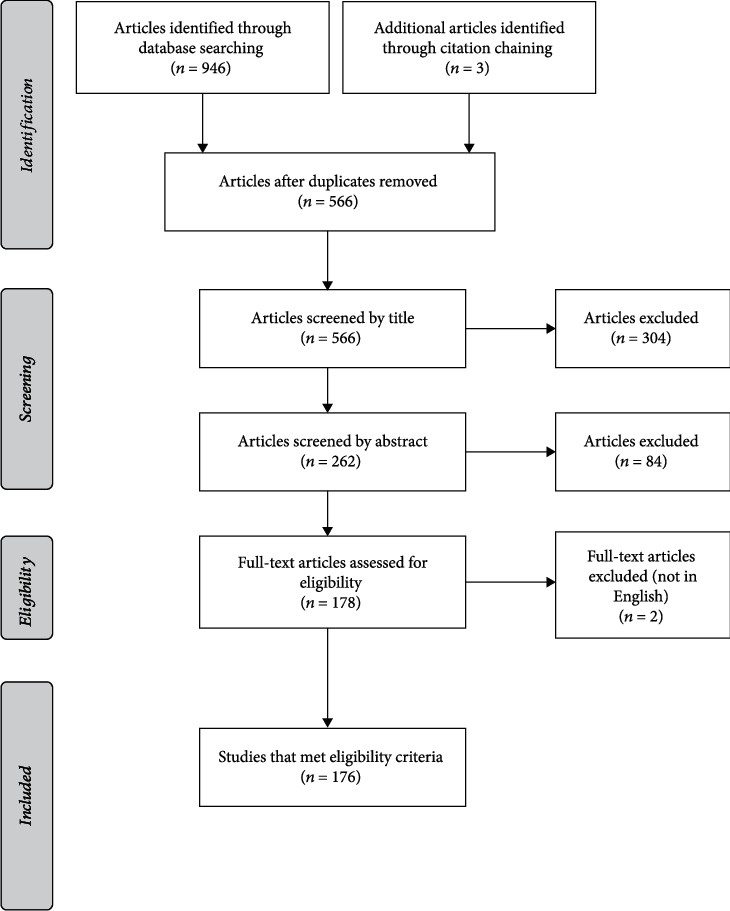
Flow chart depicting the methods used to curate this literature review.

A search of PubMed, MEDLINE, and Embase databases were conducted between June 2020 and December 2021 with the following search terms: coronavirus infections, SARS-CoV-2, coronavirus, betacoronavirus, COVID-19, nCoV, renin-angiotensin system, renin-angiotensin-aldosterone system, RAAS, RAS, angiotensin-converting enzyme 2, ACE2, eye, eyelids, ocular, lacrimal, and conjunctiva. Two independent reviewers (AA and MK) collated relevant articles by screening titles and abstracts with the results compared and combined. The search was limited to English papers, but with no restriction on publication year. Furthermore, citation chaining was utilized to ensure that no landmark studies were missed. A total of 176 references were studied and analyzed (Figure 1).

## 2. Coronavirus and the Ocular Renin-Angiotensin-Aldosterone System

### 2.1. Coronavirus

Coronaviruses belong to the *Coronaviridae* family, which are a large group of single-stranded RNA viruses that cause disease in animals and humans. They can be further classified into four genera: *Alphacoronavirus* and *Betacoronavirus* which infect mammals while *Gammacoronavirus* and *Deltacoronavirus* primarily infect birds and pigs [1]. There are seven known coronaviruses which infect humans, four of which cause mild respiratory symptoms. The other three are responsible for extensive morbidity and mortality as a result of global pandemics: Severe Acute Respiratory Syndrome (SARS) of 2002-03, Middle Eastern Respiratory Syndrome (MERS), and the recent Coronavirus Disease of 2019 (COVID-19) [1].

COVID-19 was declared a pandemic by the World Health Organization on the 11^th^ of March 2020. As of 10^th^ December 2021, over 267 million people have been infected and over 5 million deaths have been attributed to the virus [2]. With its global spread, a concentrated effort has gone into research of the responsible virus. SARS-CoV-2 is a spherical, enveloped virus that comprises of four structural proteins: surface, membrane, envelope, and nucleocapsid proteins. The surface protein is fundamental for the pathogenicity of the virus as it is involved in host cell binding. Angiotensin-converting enzyme 2 (ACE2) is the human surface receptor that is utilized by SARS-CoV-2 to facilitate infection [3].

### 2.2. Mechanisms of Infection in SARS-CoV-2

The initial presage of COVID-19 was released in late December of 2019 by an ophthalmologist, Dr. Li Wenliang. He warned about the possibility of a new SARS-like virus [4] after seeing patients with SARS-like symptoms at Wuhan Central Hospital. He later described becoming infected by SARS-CoV-2 after managing a patient with glaucoma in January 2020. Several COVID-19 cases displaying ocular symptoms have been recorded [5] instructing attentive observers about the possible ocular entry point and tropism that SARS-CoV-2 displays, facilitating respiratory but also intrinsic eye disease as well. The molecular and anatomical mechanisms of SARS-CoV-2 infection will be reviewed in the following sections.

#### 2.2.1. Receptor Mechanisms of Infection

**Figure 2:**
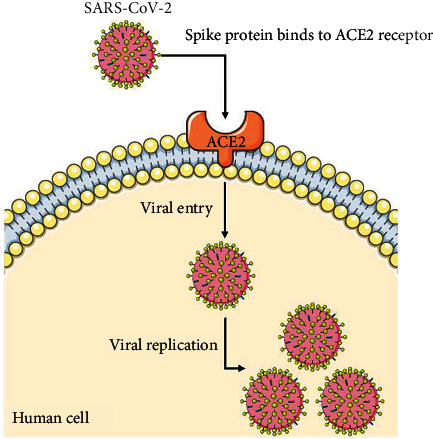
SARS-CoV-2 utilizing the ACE2 receptor located on human cells to mediate entry and replication.

The SARS-CoV-2 virus, like other betacoronaviruses, utilizes the ACE2 receptor on the surface of host cells for invasion and cellular entry (Figure 2). This has been demonstrated in virus infectivity studies using HeLa cells, where cells not expressing ACE2 were not infected by SARS-CoV-2, while cells that did express ACE2 were infected [6]. The receptor-binding domain of the spike protein located on the viral surface mediates binding to the ACE2 receptor. This occurs after priming of the spike protein by transmembrane protease, serine 2 (TMPRSS2). In fact, the binding of SARS-CoV-2 to ACE2 appears to have greater affinity compared to SARS-CoV which may contribute to the higher reproductive number (*R*_0_) of SARS-CoV-2, that is, more susceptible individuals are being infected from a single confirmed COVID-19 patient [7, 8].

#### 2.2.2. Ocular ACE2 and RAAS Components

The ACE2 receptor, a type 1 membrane-bound glycoprotein, is a crucial pivot between the classical and protective RAAS axes. It is highly expressed in vascular tissue, where it downregulates the RAAS by degrading angiotensin II (Ang II) to Ang (1–7). This has been identified to a lesser extent in numerous other organs as well. The presence of ocular ACE2 (Table 1) has been confirmed in low concentrations in the conjunctiva and cornea (especially their superficial layers [9]), the limbus, aqueous humour, and retina [10–13].

Since the discovery of renin-like activity in canine neurological tissue independent of renal renin in 1971, the local production and activity of RAAS constituents have been detected in various tissues including the eye [14]. The main components of both axes have been found across all ocular tissues, as detailed in Table 1. However, the role of local RAAS on ocular diseases and its interplay with systemic RAAS remains unclear. Currently, ocular RAAS has been implicated in diabetic retinopathy, age-related macular degeneration, retinopathy of prematurity, cataract, uveitis, glaucoma, and more recently, SARS-CoV-2 [10].

#### 2.2.3. Anatomical Mechanism of Infection

The eyes are anatomically located to provide a maximal field of view allowing for perception of the environment. However, there is an inherent vulnerability with its positioning and interconnectedness with the respiratory system, increasing susceptibility to airborne viruses. Despite being small organs, they have considerable surface areas, with several studies estimating a total palpebral aperture area between 226 and 640 mm^2^ [37, 38]. Additionally, the periorbital region also needs to be considered as it provides further area for virions to land and then migrate into the eye. This phenomenon is often witnessed when cosmetics applied around the eye drift to the eye's surface, possibly due to the action of Riolan's muscle [39]. Furthermore, the eyelids, which are responsible for keeping out foreign bodies from the eyes, may actually not be as protective to viral entry. Application of ointments on the eyelid skin, which is then transported to the eye, has been shown to be an efficient treatment modality for dry eye syndrome and this adds to the possibility of viral migration from eyelid skin to the eye. Overall, the cumulative area of the ocular surface, the eyelid skin, and the periorbital region approximately equates to 10,000 mm^2^, which is twice the area of the nares and mouth [39].

**Figure 3:**
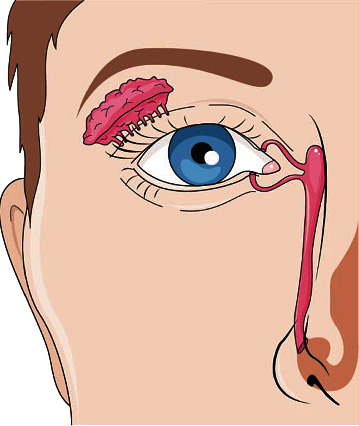
Anatomy of the nasolacrimal duct depicting a conduit between the eyes and the nose, and therefore the respiratory system.

Not only would a large surface area contribute to viral eye infection but it has also been shown that the anatomical connection between the eyes and the respiratory system, via the nasolacrimal ducts (Figure 3), can facilitate pathogenic spread to the lungs and gastrointestinal system [40].

**Figure 4:**
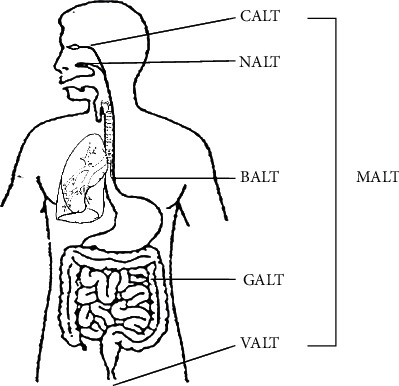
Mucosal-associated lymphoid tissue (MALT) are organised lymphoid structures including the conjunctiva (CALT), bronchus (BALT), nose (NALT), gut (GALT), and vulvovaginal (VALT).

Furthermore, the conjunctiva forms part of the organised mucosal-associated lymphoid tissues (Figure 4) known as conjunctiva-associated lymphoid tissue [41]. The lymphoid follicles of the conjunctiva ensure detection and presentation of antigens and generate an immune response that drains into the nasal-associated lymphoid tissue [41] which is continuous with lung mucosa, highlighting the compelling relationship between the respiratory system and the ocular surface. Thus, the anatomical structure of the eyes and surrounding structures may contribute to ocular and systemic disease.

### 2.3. Ocular Symptoms in COVID-19

The primary ocular finding of COVID-19, although uncommon, has been described as mild conjunctivitis, similar to that caused by other viral aetiologies. There have been other features also reported including unilateral/bilateral conjunctival hyperaemia, chemosis, epiphora, and mild eyelid oedema [42]. However, numerous studies and case series are being published demonstrating that unlike the widespread respiratory disease that the COVID-19 pandemic is causing, the occurrence of ocular symptoms is limited [43–45]. Despite the aforementioned theoretical risk of infection due to the anatomy of the eye and its adnexa and presence of ACE2 receptors on the ocular surface, the epidemiological data indicates that there may be other factors which influence the acquisition of ocular disease, discussed in the following.

#### 2.3.1. ACE2 Receptor Density

A consideration of SARS-CoV-2 infection is the location and density of ACE2 receptors. As described above, the portal of entry of the virus is through the ACE2 receptor, thus the inference can be made that the greater the number of ACE2 receptors, the increased susceptibility one has to viral entry and therefore infection. Examining the demographics of COVID-19 patients, there is a predominance of adult patients with only a minority of patients being young children, with any affected children showing less severe disease. A study investigating the levels of *ACE2* gene expression in the nasal epithelia of individuals ages 4-60 years revealed that there exists an age-dependent expression of the *ACE2* gene with younger children (4-9 years) having the lowest levels [46].

Moreover, it has been revealed that the concentration of ACE2 receptors in the conjunctival epithelial cells is less than that in the lungs [47], a possible explanation for the lower occurrence of ocular symptoms.

#### 2.3.2. Protective Properties of Tears

Tears have an important immunological role as they contain antimicrobial agents including lactoferrin and immunoglobulin A, which protect the eyes from infection by neutralizing pathogens. In addition, the flow of tears across the eye's surface into the nasolacrimal duct may serve as a mechanism of directing the SARS-CoV-2 virus away from the eyes and into the nose and lungs, possibly contributing to the low ocular and high respiratory symptoms [44]. This phenomenon was also confirmed in an animal study of cynomolgus macaque monkeys inoculated with SARS-CoV-2 via different potential routes of infection (conjunctival, tracheal, and gastric) [48]. The results revealed that the monkeys inoculated via the conjunctiva had a detectable load in the conjunctiva for the first day after inoculation, with subsequent detection in the nasal mucosa from days one to seven [48]. Therefore, the flow of tears from the eyes to the nose may be a possible route of respiratory infection, and an inherent protection mechanism against ocular infection.

#### 2.3.3. SARS-CoV-2 and the Inflammatory Response

An important consideration is the immune response to a virus and the degree of inflammation that is initiated to eliminate it. A study analyzed the innate host response to SARS-CoV-2 and compared it to other viruses including the MERS-CoV virus, the H1N1 virus causing the 2009 influenza pandemic, and the H5N1 virus causing the highly pathogenic avian influenza. It found that SARS-CoV-2 resulted in fewer proinflammatory cytokine production in *in vitro* cultures of human alveolar epithelial cells than the aforementioned viruses [49].

Additionally, a recent systematic review demonstrated that SARS-CoV-2 causes a unique cytokine response when compared with other respiratory viruses, where the often-implicated cytokines (IL-2, IL-10, IL-4, or IL-5.) are not a driving feature against the virus [50].

It is possible that these characteristics of the virus may account for low ocular symptoms, but this is yet to be proven with targeted studies on conjunctival and corneal cell samples.

### 2.4. Downregulation of ACE2 Expression/SARS-CoV-2's Interaction with the RAAS

As mentioned, the ACE2 receptor converts Ang II into angiotensin (1–7), a peptide that has the opposite functioning of Ang II, exerting a counterbalancing effect through vasodilation and antiproliferation. Indeed, there is a growing body of evidence demonstrating the protective effects it has on many pathologies such as hypertension and diabetic retinopathy [51–53].

The utilization of the ACE2 receptor by SARS-CoV-2 is not innocuous, and there is evidence that its interaction with this receptor may have a crucial effect on the RAAS. A study examined the pathogenesis of SARS-CoV and the development of acute lung injury through the virus' interaction with the ACE2 receptor [54]. It revealed that the virus downregulated the ACE2 receptor and this exaggerated lung failure due to the disturbance caused to the RAAS [55]. It was hypothesized that the decreased expression of the ACE2 receptors results in a local accumulation of Ang II which exerts its proinflammatory effects, augmenting lung injury. This finding may be translated to the SARS-CoV-2 virus since it utilizes the same receptor for cellular entry. There is a need for further research to determine its pathogenesis, helping to elucidate possible treatment modalities. If this finding is true for the SARS-CoV-2, it could mean that patients suffering from COVID-19, displaying ocular symptoms, who also have comorbid eye disease such as diabetic retinopathy may experience a worsening of their eye disease as the protective axis of the RAAS is suppressed, albeit transiently while they have the infection. However, this is likely not an implication for the majority of ophthalmic patients given the low prevalence of ocular symptoms, likely due to the aforementioned protective mechanisms.

### 2.5. Influence of Ethnicity, Age, and Sex on the Acquisition, Severity, and Morbidity of SARS-CoV-2

Ethnicity, age, and gender have been increasingly recognised to have an impact on the acquisition, severity, and morbidity of SARS-CoV-2 due to the central importance of the ACE2 receptor in enabling COVID-19 [56–60].

Significant variation in incidence and severity of COVID-19 have been documented across various ethnic groups. The UK census results indicated, after controlling for demographic and socioeconomic factors, that Black males are 4.2 times more likely to die from a COVID-19-related death than White males and Black females are 4.3 times more likely to die than White females. Furthermore, Bangladeshi, Pakistani, Indian, and mixed ethnicities were also at a statistically significant higher risk of death than White ethnicity [61]. A systematic review demonstrated that Black, Asian, and minority ethnicity patients were at a higher risk of acquiring SARS-CoV-2 infection (with Black ethnicity being the highest), and preprint literature suggested that Black and Asian ethnicities were at an increased risk of hospitalisation, admission to intensive care units, and death [59].

A potential link between SARS-CoV-2 and ethnicity has been proposed, which relates to the genetic variations and expression of ACE2 levels [59]. The importance of genetic variations has been further confirmed in the COVID-19 Chinese Han population, with overexpression of *ACE2* [60] and lower AA genotype, and A allele frequencies of *CD86 rs1129055*^9^ being linked to ARDS, and sepsis, respectively. A recent study on lung cells via single-cell RNA sequencing also found higher ACE2 pulmonary levels in Asian than White and African American donors [60]. Thus, the described polymorphisms and genetic variations in *ACE2* expression levels can likely affect the binding affinity and infection rate of SARS-CoV-2 to the human ACE2 protein [62] proffering an explanation to the differing epidemiological COVID-19 findings across ethnicities.

The severity of SARS-CoV-2 has been documented to be worse in males than females. A study examining the ACE2-expressing patterns found a higher cell ratio in males compared to females (1.66% vs. 0.41% of all cells, *p* = 0.07) [60]. Additionally, the distribution of ACE2 was more prevalent in male donors than females (5 types of cells in male lung with this receptor vs. 2-4 types of cells in female lung cells) [60]. Epidemiology studies have demonstrated that males are slightly more affected but greatly skewed in terms of severe illness and fatality. An analysis conducted early in 2020 demonstrated that of the 44,672 confirmed patients, males represented 51.4% of the total patients but 63.8% of the deaths [56]. Patients older than 60 years tend to develop more severe COVID symptoms and critical complications from the disease [56].

### 2.6. Potential Treatment Options Using the RAAS

Currently, there are no treatment modalities which take advantage of SARS-CoV-2's dependence on the ACE2 receptor for host cell entry. Blocking this mode of entry may be a potential therapeutic advancement and may curtail disease propagation within society. It appears investigation of drugs with an anti-ACE2 mechanism of action is limited. For the treatment of SARS, N-(2-aminoethyl)-1-aziridine-ethanamine, a small peptide which inhibits the activity of ACE2 was developed, but there was concern about its narrow spectrum of activity and probable effects on the RAAS function [63]. Theoretically, blocking ACE2 may result in an increase in local Ang II levels leading to inflammation and fibrosis since the classical RAAS pathway is being favoured by restricting the alternative pathway to carry out its function since ACE2 is blocked. The proinflammatory and profibrotic characteristics of the classical pathway have been extensively studied and demonstrated in other disease states such as interstitial lung disease, liver fibrosis and portal hypertension, and myocardial fibrosis [64–66].

However, there have been no laboratory or clinical studies conducted to verify this concern. Thus, focused attention to the cultivation and development of drugs which block ACE2 receptors to limit SARS-CoV-2 entry but ensure host safety may be productive. This may then be used as both treatment and prophylaxis to prevent disease transmission from both respiratory and ocular routes.

As of December 10th, 2021, there are 137 vaccine candidates that are undergoing research and testing in human trials and 194 candidates in preclinical development [67], but none focus on the RAAS pathway in its mode of action. Numerous techniques have been utilized to develop vaccines that facilitate host immune response and confer secondary immunity, including mRNA and DNA technology, viral vectors (replicating and nonreplicating), inactivated viruses, live attenuated viruses, and protein subunit vaccines [68]. The main three vaccines currently available are the Pfizer/BioNTech vaccine, the Moderna vaccine, and the AstraZeneca/Oxford vaccine.

The Pfizer/BioNTech vaccine (BNT162b2) has the most emergency use approvals from countries including the UK, Canada, and the US and demonstrates a 95% efficacy in preventing COVID-19 [69]. It utilizes mRNA enclosed in a lipid carrier to stimulate the production of host memory B and T cells to the SARS-CoV-2 spike protein. In addition to reducing the number of subjects infected with COVID-19, it also reduces the severity of symptoms for those vaccinated. This vaccine needs to be kept at -70 degrees Celsius for long term storage, which poses its own unique challenges in the global setting [70].

The Moderna vaccine also utilizes mRNA contained within a phospholipid nanoparticle and has received emergency use authorisation in the US. Unlike the Pfizer/BioNTech vaccine, the Moderna vaccine has more pragmatic storage conditions with the vaccine being able to be stored at 2-8 degrees Celsius for up to thirty days [71]. It has shown efficacious results in the phase III trial with 94.1% reduction in COVID-19 symptoms in those who received the vaccine [72].

The AstraZeneca/Oxford vaccine has a different method of priming host defences. It uses a chimpanzee adenovirus to insert DNA coding for the SARS-CoV-2 spike protein into host cells so that translational and transcriptional enzymes can convert these genetic instructions into the spike protein for display on MHC class I/II, generating memory B and T cells [73]. It has similar storage requirements to the Moderna vaccine (2-8 degrees Celsius) but has lower reported efficacy of 70% [74].

Despite these vaccines and the estimation that by the end of 2021 there will be billions of vaccine doses ready for administration worldwide [75], the current best practice for disease prevention still remains social distancing amongst community members and isolation of COVID-19 patients [76].

## 3. Conclusion

Despite the presence of ACE2 on various ocular structures, the incidence of COVID-19-associated eye disease remains low. This may be explained by the differing ACE2 receptor densities in the eyes as compared to the lungs coupled with the protective property of tears and the intrinsic host inflammatory response being milder and orchestrated by different cytokines targeted against SARS-CoV-2 as compared to other respiratory viruses. These suppositions need to be confirmed with studies on conjunctival and corneal cell samples.

## Figures and Tables

**Table 1:** Locations in the eye where RAAS components can be detected. Highlighted cells represent the presence of RAAS component within the particular human ocular structure.

	Tears/lacrimal gland	Bulbar conjunctiva	Cornea	Sclera	Aqueous humour	Iris	Trabecular meshwork	Ciliary body/nonpigmented ciliary epithelium	Vitreous	Choroid	Retina	Optic nerve head	References
Prorenin		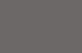	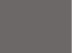	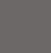	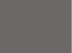	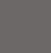		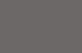	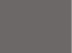				[15–19]
Renin		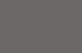	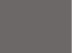	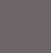	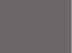	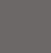		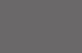	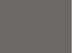	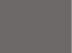	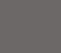		[15, 18, 19]
Angiotensinogen		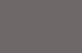	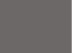	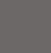	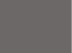	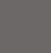		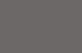	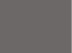	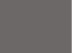	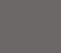		[15, 20–23]
Angiotensin I					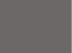	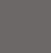		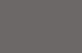	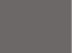				[18, 24]
Angiotensin II		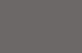	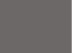		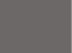	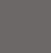	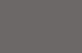	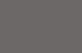	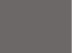	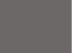	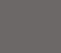	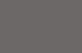	[18, 24–26]
Angiotensin 1-7					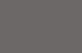		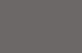	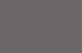			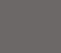		[26–28]
ACE1	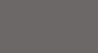	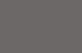	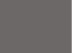	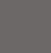	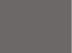	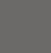	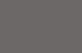	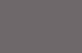	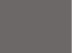	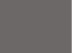	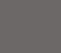	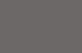	[15, 21, 25, 28–35]
ACE2		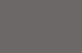	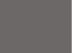		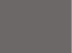								[26, 36]

## Data Availability

The data supporting this review article are from previously reported studies and datasets, which have been cited.

## Abbreviations

ACE2: Angiotensin-converting enzyme 2
Ang II: Angiotensin II
COVID-19: Coronavirus disease of 2019
MERS: Middle Eastern Respiratory Syndrome
RAAS: Renin-angiotensin-aldosterone system
RNA: Ribonucleic acid
SARS: Severe Acute Respiratory Syndrome
TMPRSS2: Transmembrane protease, serine 2.
